# Association between electronic cigarette use and depression among Thai adolescents: The Thailand National Health Examination Survey 2019–2020

**DOI:** 10.18332/tid/155333

**Published:** 2022-11-18

**Authors:** Roengrudee Patanavanich, Patriya Vityananan, Nareemarn Neelapaichit, Suwat Chariyalertsak, Pattapong Kessomboon, Sawitri Assanangkornchai, Surasak Taneepanichskul, Wichai Aekplakorn

**Affiliations:** 1 Department of Community Medicine, Faculty of Medicine, Ramathibodi Hospital, Mahidol University, Bangkok, Thailand; 2 Ramathibodi School of Nursing, Faculty of Medicine, Ramathibodi Hospital, Mahidol University, Bangkok, Thailand; 3 Faculty of Public Health, Chiang Mai University, Chiang Mai, Thailand; 4 Faculty of Medicine, Khon Kaen University, Khon Kaen, Thailand; 5 Department of Epidemiology, Faculty of Medicine, Prince of Songkla University, Songkhla, Thailand; 6 College of Public Health Sciences, Chulalongkorn University, Bangkok, Thailand

**Keywords:** Thailand, adolescent, depression, e-cigarette

## Abstract

**INTRODUCTION:**

Depression and e-cigarette use among adolescents are two health burdens. However, the association between these dual problems have been less studied, especially in low- and middle-income countries. This study examined the association between depression and e-cigarette use among adolescents in Thailand.

**METHODS:**

This cross-sectional study used the sub-sample of the sixth Thai National Health Examination Survey conducted between 2019 and 2020. A total of 4237 adolescents aged 10–19 years were included. Self-reported depression was captured using the 20-item Centre for Epidemiologic Studies Depression Scale (CES-D). We applied a complex survey multiple logistic regression to assess whether e-cigarette use was associated with depression.

**RESULTS:**

The mean age of the participants was 14.6 years, 5.3% were ever e-cigarette users, and 2.9% were current e-cigarette users. 37.8% of the participants were categorized at risk for depression. Among e-cigarette users, 51.6% of ever e-cigarette users and 52.9% of current e-cigarette users were at risk for depression. Multiple logistic regression revealed that ever e-cigarette users were at higher risk for depression (AOR=1.66; 95% CI: 1.02–2.71; p=0.042) than never e-cigarette users. Current e-cigarette was not associated with a higher risk for depression (AOR=1.37; 95% CI: 0.77–2.45; p=0.263).

**CONCLUSIONS:**

E-cigarette use and depression among adolescents are global public health concerns. There is also a need for effective screening, prevention, and intervention to reduce adverse outcomes of e-cigarette use and depression. In addition, the government should strengthen current policies and close legal loopholes to prevent the tobacco industry tactics and keep e-cigarettes away from adolescents.

## INTRODUCTION

Depression is a common mental disorder among adolescents aged 10–19 years with a global total of over 4.2 million years lived with disability (YLD) in 2019, a 26% increase from 1990^1^. Between 2001 and 2020 the global point prevalence rate of elevated self-reported depressive symptoms among adolescents aged 10–19 years was 34%, which exceeds the reported estimates for young adults^2^. The higher rates of depression among adolescents occurred simultaneously when novel and emerging tobacco products became popular, raising the question regarding whether depressive symptoms were associated with electronic cigarette (e-cigarette) use^3^.

Associations between conventional cigarette smoking and depression among adolescents have been well documented^4,5^. However, research on e-cigarettes and depression is insufficient. According to a recent systematic review, only seven studies examined associations between e-cigarette use and depressive symptoms among adolescents^6^. All studies were conducted in high-income countries, including US (4 studies), Korea (2 studies), and Taiwan (1 study). Although most studies suggested positive relationships, there were some limitations due to cross-sectional designs, single-item measures of depression, and minimal adjustment for confounders^5^. There was no study conducted in low- or middle-income countries (LMICs).

Thailand, an LMIC, is among 32 countries worldwide that strictly prohibits e-cigarettes^7^. Despite the ban, e-cigarettes are available, especially over online platforms and flea markets due to legal loopholes and weak law enforcement^8^. The prevalence of e-cigarette use among Thai adolescents (aged 13–15 years) increased from 3.3% in 2015 to 8.1% in 2021^9^. The popularity of e-cigarettes raises concerns about the significant health risks these products pose to teens. Coincidently, a survey in 2021 found that 32% of Thai adolescents were at risk of depression and 22% had suicidal ideation^10^. Thus, this study explored whether e-cigarette use was associated with depression and the risk of clinical depression among Thai adolescents.

## METHODS

### Data source

This study was a cross-sectional study based on the sixth Thai National Health Examination Survey (NHES-VI), which was a nationally representative survey using multistage, stratified sampling of the Thai population aged ≥10 years. The NHES is the largest cross-sectional, non-institutionalized population representative survey in Thailand, completed every five years^11^. A detailed description of the Thai NHES series has been published elsewhere^12^. The NHES-VI was conducted from August 2019 to October 2020 with 32400 participants, yielding a response rate of 92.2%. For this study, 4237 adolescents aged 10–19 years from the NHES-VI survey were included.

### Measures

*Depression*

Self-reported depression was captured using the 20-item Centre for Epidemiologic Studies Depression Scale (CES-D)^12^. Each item is scored on a 4-point Likert scale (0–3) based on the frequency of occurrence of the symptom^13^. Total scores range from 0 to 60, with higher scores indicating greater depressive symptoms^13^. A cut-off score of ≥16 was typically recommended^14^ and thus used in this study to identify individuals at risk for clinical depression.

*E-cigarette use*

Ever e-cigarette use was identified if the respondent answered ‘yes’ to the question: ‘Have you ever used an e-cigarette, even just one time?’. Current e-cigarette use was assessed by the question: ‘During the past 30 days, on how many days did you use electronic cigarettes?’. If the response was ≥1 day, the respondent was identified as a current e-cigarette user.

*Other covariates*

Covariates included in this study were individual (age, sex, body mass index, and health status), behavioral (current alcohol drinking), socioeconomic (parental college education, good relationship with parents, friends’ aggressive characteristics, and experiencing sexual harassment), and environmental (live near slum, live near pubs and bars, and feeling unsafe at school) risk factors.

The definition of these variables was defined as follows. Poor health status was being sick more than 4 times in the past 12 months. Current alcohol drinking was alcohol use in the past 30 days. Parental college education was identified if the adolescents’ father or mother graduated from college. A good relationship with parents was identified if adolescents said they felt close to their fathers or mothers and can ask for advice. Having aggressive friends was identified if adolescents reported at least a common characteristic of their friends (aggressive verbal communication, rude verbal communication, fighting, physically hurting others, or sneaking out). Experiencing sexual harassment was identified if adolescents had been sexually harassed (verbally or physically) in the past 12 months. Live near slums or pubs and bars was identified if adolescents lived within a 30-minute walk or 1 km from these places. The unsafe school was identified if adolescents did not go to school because of feeling unsafe for at least one day in the past 30 days.

### Statistical analysis

We used the NHES-provided weights and stratification variables to adjust for multistage sampling design and non-response to match sample characteristics of national estimates. We computed descriptive statistics of the adolescents with and without depression based on socioeconomic characteristics and used weighted chi-squared statistics (for categorical variables) or independent t-tests (for continuous variables) to compare the differences. Associations between use of e-cigarettes and depression were computed using multiple logistic regression accounting for the complex survey design and adjusting for other risk factors. The statistical analysis was conducted using the ‘*svyset*’ command in STATA 14^15^ and a p<0.05 was considered statistically significant.

## RESULTS

The mean age of the participants was 14.6 years. The proportion of male participants was slightly higher than females (53.0% vs 47.0%). Based on the summary score of CES-D (≥16), 37.8% of the participants were categorized at risk for depression; 5.3% of the participants were ever e-cigarette users, 2.9% were current e-cigarette users, and 26.1% were current alcohol users. Among e-cigarette users, 51.6% of ever e-cigarette users and 52.9% of current e-cigarette users were at risk for depression. Adolescents with depression were more likely to be female, overweight, have poor health status, e-cigarette users, alcohol drinkers, have poor relationship with parents, have aggressive friends, experience sexual harassment, live near pubs and bars, and feel school is unsafe. Characteristics of the study population can be found in Table 1.

**Table 1:** Depression of adolescents by characteristics, from the sixth Thai National Health Examination Survey (NHES-VI), 2019–2020 (N=4237)

*Characteristics*	*Overall n (wt. %)*	Depression*		*p*
		*Yes n (wt. %)*	*No n (wt. %)*	
**Individual**				
**Age** (years), mean	14.6	14.7	14.6	0.448
**Sex**				<0.001
Male	2118 (53.0)	633 (46.2)	1474 (57.3)	
Female	2119 (47.0)	832 (53.8)	1270 (42.7)	
**BMI** (mean)	21.1	21.5	20.9	0.002
**Health status**				0.031
Good	3837 (90.7)	1302 (89.1)	2528 (91.7)	
Poor	381 (9.3)	163 (10.9)	216 (8.3)	
**Behavioral**				
**Ever e-cigarette use**				0.002
Yes	154 (5.3)	57 (6.7)	78 (3.8)	
No	4083 (94.7)	1408 (93.3)	2666 (96.2)	
**Current e-cigarette use**				0.007
Yes	85 (2.9)	31 (3.4)	35 (1.8)	
No	4152 (97.1)	1434 (96.6)	2709 (98.2)	
**Current alcohol use**				0.017
Yes	393 (26.1)	411 (69.6)	749 (76.7)	
No	1161 (73.9)	160 (30.4)	233 (23.3)	
**Socioeconomic**				
**Parent college graduate**				0.944
Yes	516 (10.0)	183 (10.1)	332 (10.0)	
No	3721 (90.0)	1282 (89.9)	2412 (90.0)	
**Good relationship with parents**				<0.001
Yes	2869 (67.0)	934 (62.2)	1929 (70.5)	
No	1368 (33.0)	531 (37.8)	815 (29.5)	
**Have aggressive friends**				<0.001
Yes	3033 (70.6)	1140 (76.2)	1887 (67.7)	
No	1204 (29.4)	325 (23.8)	857 (32.3)	
**Experiencing sexual harassment**				<0.001
Yes	158 (4.4)	87 (7.6)	51 (1.6)	
No	4079 (95.6)	1378 (92.4)	2693 (98.4)	
**Environmental**				
**Live near slum**				0.015
Yes	316 (7.0)	127 (8.4)	189 (6.2)	
No	3899 (93.0)	1336 (91.6)	2554 (93.8)	
**Live near pubs and bars**				0.047
Yes	262 (7.2)	108 (8.7)	154 (6.3)	
No	3953 (92.8)	1355 (91.3)	2589 (93.7)	
**Feel school unsafe**				<0.001
Yes	286 (5.9)	135 (7.5)	131 (4.2)	
No	3951 (94.1)	1330 (92.5)	2613 (95.8)	

* Self-reported depression was captured using the 20-item Centre for Epidemiologic Studies Depression Scale (CES-D) with a cut-off score of ≥16. BMI: body mass index (kg/m^2^).

The multiple logistic regressions (Table 2) revealed that ever-e-cigarette users were at higher risk for depression when compared to never e-cigarette users (AOR=1.66; 95% CI: 1.02–2.71; p=0.042). Current e-cigarette use also yielded an elevated odds ratio of higher risk for depression but it did not reach the level of statistical significance (AOR=1.37; 95% CI: 0.77–2.45; p=0.263). Other factors associated with an increased odds of depression were female gender (AOR=2.17; 95% CI: 1.01–1.98; p=0.043), BMI (AOR=1.03; 95% CI: 1.01–1.05; p=0.003), current alcohol drinking (AOR=1.42; 95% CI: 1.01–1.05; p=0.003), having a parent a college graduate (AOR=1.38; 95% CI: 1.06–1.80; p=0.002), having aggressive friends (AOR=1.61; 95% CI: 1.20–2.16; p=0.004), and experiencing sexual harassment (AOR=4.03; 95% CI: 2.20–7.37; p<0.001). A good relationship with parents was more likely to decrease the odds of depression (AOR=0.60; 95% CI: 0.48–0.75 ; p <0.001).

**Table 2:** Multiple logistic regression between e-cigarette use and high risk for depression among adolescents from the sixth Thai National Health Examination Survey (NHES-VI), 2019–2020 (N=4237)

*Characteristics*	*Risk of depression Model 1*			*Risk of depression Model 2*		
	*AOR*	*95% CI*	*p*	*AOR*	*95% CI*	*p*
**Individual**						
Age (per year increase)	0.97	0.86–1.10	0.609	0.97	0.87–1.10	0.656
Female	2.17	1.75–2.68	<0.001	2.09	1.71–2.55	<0.001
BMI (per 1 kg/m^2^ increase)	1.03	1.01–1.05	0.003	1.03	1.01–1.05	0.004
Poor health status	1.07	0.69–1.66	0.747	1.07	0.69–1.66	0.764
**Behavioral**						
E-cigarette use						
Ever e-cigarette use	1.66	1.02–2.71	0.042			
Never e-cigarette use (Ref.)	1					
Current e-cigarette use				1.37	0.77–2.45	0.263
Non-e-cigarette use (Ref.)				1		
Current alcohol use	1.42	1.01–1.98	0.043	1.49	1.04–2.14	0.031
**Socioeconomic**						
Parent college graduate	1.38	1.06–1.80	0.02	1.35	1.04–1.77	0.029
Good relationship with parents	0.60	0.48–0.75	<0.001	0.60	0.48–0.74	<0.001
Have aggressive friends	1.61	1.20–2.16	0.004	1.63	1.23–2.18	0.002
Experiencing sexual harassment	4.03	2.20–7.37	<0.001	4.08	2.25–7.40	<0.001
**Environmental**						
Live near slum	1.38	0.91–2.09	0.121	1.39	0.92–2.12	0.113
Live near pubs and bars	0.91	0.57–1.46	0.68	0.93	0.57–1.50	0.745
Feel school unsafe	1.33	0.79–2.23	0.267	1.33	0.78–2.25	0.272

AOR: adjusted odds ratio. Model 1 explored a relationship between ever e-cigarette use and depression, adjusted for all covariates. Model 2 explored a relationship between current e-cigarette use and depression, adjusted for all covariates.

## DISCUSSION

The present study examined the association between e-cigarette use and depression among Thai adolescents at the population level. In unadjusted analyses, we found that both ever and current e-cigarette use were significantly associated with depression. However, when adjusted for other covariates, only ever e-cigarette use was significantly associated with depression. A possible explanation could be the relationship between e-cigarettes and depression is bi-directional^3^. Some teens who try e-cigarettes to relieve stress may not become regular users due to less availability of e-cigarettes in Thailand when compared to countries with less restrictive policies^7^. Furthermore, the results of non-significant association were limited by the small sample of current e-cigarette users.

Similar to previous studies in high-income countries^6^, our results revealed additional evidence to establish a positive association between e-cigarette use and depression among adolescents in an LMIC. The association between e-cigarette use and depression among adolescents is critical and could have potentially significant public health implications. Previously, the World Health Organization (WHO) called a relationship between tobacco use and mental health problems ‘a double burden on health’^16^. Some researchers believe the relationship between smoking and depression is bi-directional^5^; however, Mendelian randomization studies confirmed a causal relationship between smoking and depression – that is, smoking is a risk factor for depression^17-19^. Although the physiological connection between smoking and depression is less known, numerous peer-reviewed articles discovered that nicotine can worsen symptoms of depression^20^. A salient symptom of nicotine dependence is withdrawal, which can exacerbate mental health illnesses, including depression^20^. Moreover, a recent study revealed that daily exposure to flavored nicotine e-cigarettes may induce neuro-inflammation in brain regions responsible for the formation of depressive behaviours^21^. Further research is needed to fully understand the relationship between e-cigarette use and depression, whether it is a bi-directional or causal relationship.

More concern about e-cigarette use and depression among youth is expected, as e-cigarette companies use various marketing tactics to target youth^22^. In addition, the wide variety of flavored nicotine e-cigarette products hook youth more quickly, as these products offer the user a far greater range of attractive tastes and sensory experiences combined with the advent of the use of protonated nicotine that enhances nicotine delivery^23,24^. These tactics seem to work as e-cigarette use among youth has become a global epidemic. Additionally, the tobacco industry consistently spreads misleading messages that nicotine relieves stress, anxiety, and depression^25^. Not surprisingly, stress relief was the most commonly cited reason for vaping among adolescents without knowing that it could worsen their mental health concerns^26^. E-cigarettes are quite new and a large number of people, especially in LMICs, are unaware of them and their health risks^27^. This is a public health challenge to promote comprehensive educational campaigns about the risks of e-cigarettes ahead of the tobacco industry’s tactics to recruit new users. Healthcare providers should become more aware of the health effects of e-cigarettes and the tactics of the tobacco industry to promote e-cigarettes among youth.

Consistent with previously identified risk factors for depression, we also found that biopsychosocial risk factors such as being female, obesity, alcohol drinking, peer problems, and victims of sexual harassment, were associated with depression as these factors negatively affect individual self-esteem^28,29^. On the other hand, the strong family relationship is a protective factor against youth depression^30^. Clinicians should also be aware of these comorbidities when providing care for adolescents. Moreover, mental health screening for adolescents who have a history of using e-cigarettes should be recommended in all healthcare settings and providers should always ask about e-cigarette experiences when treating depressive adolescents.

### Strengths and limitations

Our study has several limitations. Because this study relies on a cross-sectional study design, the findings may not reflect a causal relationship between e-cigarettes and depression. The questionnaire is lengthy and based on self-reported data; thus, could be subject to reporting errors. In addition, due to the lack of information available on cigarette or other nicotine use, we do not know if the e-cigarette users were dual users, and cannot directly attribute the association of depression to e-cigarette use. However, we included a variety of potential confounders, including individual, behavioral, social, and environmental risks. This study has other strengths. To our knowledge, this is the first study in LMICs to demonstrate a relationship between e-cigarette use and depression among adolescents. Our study also relies on the most recent national representative data for adolescents in Thailand. Also, rather than using single-item measures of depression, this study employs CES-D, which has demonstrated reliability and validity for assessing depression among adolescents^31,32^.

## CONCLUSIONS

E-cigarette use and depression among adolescents are double public health burdens that the government and public health sectors should be aware of. The dual problems do not exist only in high-income countries, but also impact LMICs, even where e-cigarettes are prohibited. Thus, strengthening current policies and closing legal loopholes to prevent tobacco industry tactics and keeping e-cigarettes away from adolescents, should be a priority. There is also a need for effective screening, prevention, and intervention to reduce adverse outcomes of depression.

## Data Availability

The data supporting this research are available from the authors on reasonable request. All data used to prepare this article are available from the cited sources. The sixth Thai National Health Examination Survey (NHES-VI) can be requested directly from the NHES Project, Faculty of Medicine Ramathibodi Hospital, Mahidol University, Bangkok, Thailand.
